# Gut check: Exploring tools, techniques, and future directions in microbiome research

**DOI:** 10.1016/j.psj.2026.106713

**Published:** 2026-02-27

**Authors:** Samson Oladokun, Bertrand Grenier, Brian Oakley, Cristiano Bortoluzzi, Mahalingam Ramkumar

**Affiliations:** a Department of Poultry Science, Texas A&M University, College Station, TX 77843, USA; b dsm-firmenich, Village-Neuf, France; c Zinpro Corporation, Eden Prairie, MN, USA; d College of Veterinary Medicine, Western University of Health Sciences, Pomona, CA 91766, USA; e Computer Science & Engineering, Mississippi State University, Starkville, MS 39762, USA

**Keywords:** Microbiome, Gut, Next-Generation Sequencing, Deep Learning Models, Poultry

## Abstract

Poultry microbial communities are now recognized as key contributors to host nutrition, immune function, disease resilience, and overall health and performance, driving growing interest in this research field. This symposium brought together leading experts to discuss the latest advancements in analytical tools and technologies for investigating the poultry microbiome. Presentations highlighted current progress in microbiome profiling techniques, next-generation sequencing technologies, advanced data analysis methods such as machine learning, and the integration of cutting-edge approaches in microbiome research. A roundtable discussion further engaged participants in identifying key challenges in the field, including method standardization, reproducibility, and data interpretation, while emphasizing emerging opportunities to translate microbiome insights into practical strategies for disease control, antibiotic alternatives, and sustainable poultry production. The knowledge shared in this symposium is highly relevant to poultry researchers and the industry, as they work to enhance bird health, welfare, and productivity.

## Introduction

Interactions between poultry hosts and its microbial communities influences health outcomes, disease susceptibility, and gut functionality (Oladokun and Sharif, 2024). The study of poultry microbiomes has now emerged as one of the fastest-growing areas of avian research. According to the Poultry Science journal website, more than 650 microbiome-related studies have been published in this journal alone over the past five years. This rapid growth is driven in part by advances in next-generation sequencing technologies and a rising interest in developing effective alternatives to antibiotics for poultry production. Despite this progress, the expansion of microbiome research brings significant complexities, including wide variation in experimental design, methodology, analytical approaches, computational tools, and data interpretation. To fully realize the benefits of microbiome science, greater attention must be directed toward the refinement of tools, techniques, and future applications in poultry research.

Here, a concise overview of the historical development of poultry microbiota-profiling technologies, their potential and future applications in poultry research and industry, the challenges associated with microbiome studies, and key recommendations for advancing the field are presented. Additionally, current next-generation sequencing platforms are also discussed, alongside a practical case study of the application of deep learning models for microbiome analysis in poultry research.

### Chicken gut microbiota profiling: history and future directions

Brian Oakley (boakley@westernu.edu)

College of Veterinary Medicine, Western University of Health Sciences, Pomona, CA, United States

The taxonomic composition and metabolic functions of the gastrointestinal (**GI**) microbiota of chickens are important for nutrition, immunity, productivity, and disease resistance (Oakley et al., 2014). Because microbial genes outnumber chicken genes by >100-fold, the concept of a chicken as a “meta-organism”, in which host and microbes function as an integrated system, is important for researchers and industry to consider (Gilroy et al., 2021; Glendinning et al., 2020; Segura-Wang et al., 2021).

The GI microbiome is dynamic - shaped by age, diet, host genetics, and environmental factors such as litter, drinking water, or airborne microbes (Kers et al., 2018). Additionally, significant taxonomic and functional differences occur depending on anatomical location (Yeoman et al., 2012). Environmental stressors such as high ambient temperature further contribute to microbial community dynamics (Campos et al., 2023; Lyte et al., 2025). As for all animal-associated microbial communities, early-life colonization events are particularly important for the poultry GI microbiome, as they can set longer-term trajectories that influence health and performance over the lifespan of a bird (Ramírez et al., 2020).

Historically, the adoption of culture-independent molecular methods to characterize microbial communities was an important advance following the recognition of the so-called ‘great plate count anomaly’ (Staley and Konopka, 1985), that many microbes are difficult or impossible to grow in traditional culture media. Early microbiota profiling methods relied on molecular fingerprinting methods such as Denaturing Gradient Gel Electrophoresis (DGGE; Muyzer et al., 1993) or Terminal Restriction Fragment Length Polymorphism (**tRFLP**; Liu et al., 1997) which were labor-intensive, low-resolution, and lacked standardized reference databases. Early DNA sequencing methods were an improvement on these fingerprinting methods but could not provide adequate sampling depth to properly characterize and compare communities (Bent and Forney, 2008). The advent of high-throughput sequencing (**HTS**) - in one of three main applications - has therefore revolutionized the field. First, amplicon sequencing of 16S rRNA genes is used to provide a complete taxonomic census of a microbial community. Second, metagenomics can provide a “parts list” of microbial gene content, and third, metatranscriptomics can provide a snapshot of microbial gene expression for a particular time or experimental condition.

In modern poultry microbiology, it is now routinely possible to use one or more of these methods to improve our understanding of the mechanisms by which the GI microbiome can influence poultry production. For example, a recently published paper (Ramirez et al., 2022) used 16S rRNA sequencing and metagenomics to identify specific taxa and metabolic pathways associated with significant improvements in body-weight gain and feed efficiency. In this work, adult cecal contents were transplanted via oral gavage to day-of-hatch chicks, and provided significant improvements in performance that were localized specifically to cecal microbes. In particular, uptake hydrogenase genes from the genus *Methanobrevibacter*, associated with hydrogen consumption and fermentative production of short-chain fatty acids, were significantly over-represented in high-performing birds suggesting that hind-gut fermentation by cecal microbes can have significant effects on performance (Ramirez et al., 2022).

Despite continuing technological advances however, multiple challenges in microbiota profiling remain. For example, amplicon sequencing provides mainly taxonomic—not functional—insights, with limited resolution at the strain level. Metagenomics and metatranscriptomics yield vast datasets that can be expensive to generate and difficult to analyze. Sampling strategies also complicate interpretation as fecal and cloacal samples do not reliably represent communities in other parts of the GI tract (Oakley and Kogut, 2016; William and Athrey, 2020). Protocols for fieldwork, bench techniques, and bioinformatics also lack standardization and bioinformatics remains a key bottleneck. While sequencing costs continue to decrease, data analysis and interpretation remain resource intensive (Weinroth et al., 2022).

Looking ahead, there are needs for improved multi-omic integration, application of machine learning and artificial intelligence, and long-read sequencing. We foresee that these tools will advance the field of poultry microbiology in several areas:

• **Improving flock, human, and environmental health:** routine microbiome monitoring could enable early detection of pathogens, improve biosecurity, and integrate animal and environmental surveillance.
• **Microbiome programming**: targeted early-life interventions, synthetic microbial communities, and evidence-based probiotics all hold potential for long-term improvements to food-safety and performance.
• **Biomarker discovery**: specific taxa or genes could serve as indicators of feed efficiency, subclinical diseases (e.g., necrotic enteritis), or dysbiosis, paralleling indices already developed for companion animals.
• **Precision nutrition**: microbiota-informed diet formulation could optimize nutrient supply, linking dietary inputs with microbial metabolism to maximize productive outputs.

### Next-generation sequencing (NGS) technologies for rapid microbiome profiling

Cristiano Bortoluzzi^+^, Bertrand Grenier^++^, Samson Oladokun*

^+^dsm-firmenich, Kaiseraugst, Switzerland; Current address: Zinpro Corporation, Eden Prairie, MN, USA (cbortoluzzi@zinpro.com)

^++^dsm-firmenich, Village-Neuf, France (Bertrand.Grenier@dsm-firmenich.com)

*Department of Poultry Science, Texas A&M University, College Station, TX 77843, USA (samson.oladokun@ag.tamu.edu)

Next generation sequencing technologies (NGS) have transformed microbiome research by enabling high throughput, culture independent characterization of microbial communities. Unlike first generation approaches that focused on individual cultured isolates, NGS shifted microbiology toward community level analysis, allowing simultaneous taxonomic and functional interrogation of complex ecosystems (Crofts et al., 2017). This transition has supported advances in microbial ecology, pathogenesis, and clinical or production applications. Comprehensive descriptions of the NGS workflow, including poultry specific considerations from sampling and preservation through DNA extraction, library preparation, sequencing, bioinformatics, data integration, and data deposition, are provided by Lyte et al. (2025).

**Fig. 1:**
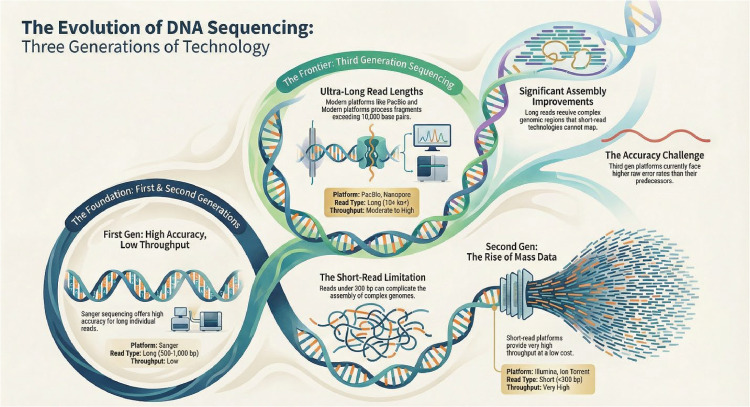
Overview of the evolution of sequencing platforms. Information adapted from Sanger et al. (1977); Voelkerding et al. (2009); Tipu et al. (2015); Van Dijk et al. (2018). (Image generated with Artificial Inteligence from NotebookLM of google).

An overview of the evolution of sequencing platforms is provided in Fig. 1. Historically, first generation sequencing was introduced by Frederick Sanger in 1977 and relied on selective incorporation of fluorescently labeled chain terminating dideoxynucleotides during polymerase chain reaction amplification (Sanger et al., 1977; Liu et al., 2012). Although the Sanger method enabled automated DNA sequencing, its low throughput and high cost limited its utility for large scale microbial community studies (Metzker, 2010). Second generation sequencing, often referred to as next generation sequencing, emerged in the early 2000s with the development of the 454 Pyrosequencer and the launch of the Genome Sequencer 20 system by 454 Life Sciences (Metzker, 2005). These systems combined emulsion PCR with sequencing by synthesis chemistry, substantially increasing throughput while reducing cost and time relative to Sanger sequencing (Díaz-Sánchez et al., 2013). Major second-generation platforms were commercialized by Roche, Illumina, Life Technologies, Helicos, and others. Despite their impact, second generation systems require complex library preparation, are susceptible to dephasing during cyclic sequencing reactions, and exhibit increasing error rates toward the ends of reads (Metzker, 2010; Schatz et al., 2010). Third generation sequencing technologies address some of these limitations by enabling single molecule sequencing without prior amplification. Representative platforms include Single Molecule Real Time sequencing developed by Pacific Biosciences and nanopore based sequencing from Oxford Nanopore Technologies. These systems generate long reads that improve genome assembly, structural variant detection, and resolution of repetitive regions. Selection of a sequencing platform must be aligned with the biological question, required resolution, acceptable error tolerance, throughput, and budget. Given the strengths and limitations of each sequencing platform (Fig. 1), researchers must carefully choose the platform that best addresses their research question. Some platforms maximize total output per run, whereas others prioritize read length, speed, or accuracy. In some cases, combining short read and long read approaches improves genome reconstruction and functional annotation.

In poultry microbiome research, 16S rRNA gene amplicon sequencing and shotgun metagenomics are the most widely applied NGS strategies (Shang et al., 2018).

#### Amplicon Sequencing

Amplicon sequencing is widely used in poultry microbiota research, most commonly on the Illumina HiSeq and MiSeq platforms (Shang et al., 2018). This approach targets and amplifies specific marker genes, typically conserved regions of the bacterial 16S rRNA gene or the fungal internal transcribed spacer region (ITS), to infer community composition. The structural conservation and interspersed hypervariable regions of the 16S rRNA gene make it suitable for bacterial identification (Weinroth et al., 2022). It has therefore been widely applied in several poultry studies (Ma et al., 2017; Pandit et al., 2018; Ocejo et al., 2019; Oladokun et al., 2021, 2022). High multiplexing capacity, relatively low per sample cost, established laboratory workflows, and available bioinformatics pipelines facilitate cross study comparisons and integration with existing literature (D’Amore et al., 2016).

However, these advantages are accompanied by methodological constraints. Taxonomic resolution is often restricted to the genus level because short hypervariable regions cannot reliably discriminate closely related species or strains. Cheaper cost mostly comes at the expense of Taxonomic resolution. The 16S rRNA gene contains nine hypervariable regions, and primer selection influences taxonomic resolution, diversity estimates, and interpretation of results (Highlander, 2012; Oladokun et al., 2021). This effect has been validated in a few poultry studies (Zhao et al., 2013; Darwish et al., 2021). García-López et al. (2020) showed that V3 and V3V4 regions capture broader diversity than the V4 region, whereas V4 primers yield profiles more similar to those obtained by shotgun sequencing (Tremblay et al., 2015). The V3 region may offer faster sequencing and lower cost, while V3V4 can provide higher taxonomic resolution at increased expense (García-López et al., 2020). PCR amplification introduces additional biases, including chimera formation, and inflated richness estimates (Abellan-Schneyder et al., 2021). Short read lengths and sequencing errors further limit resolution (Quince et al., 2009, 2011). Because only a small marker region is sequenced as opposed to the whole genome, functional potential cannot be directly measured (Jovel et al., 2016; Matchado et al., 2024).

Di Bella et al. (2013) provides a detailed discussion of pros and cons of each NGS platform. Typically, Illumina HiSeq provides high output at relatively low reagent cost per base and can generate up to 600 gigabases per run with high accuracy compared to Roche 454 (Liu et al., 2012; Luo et al., 2012; Di Bella et al., 2013). As the most commonly used NGS platform in poultry microbiome studies, the MiSeq shares core technology with the HiSeq and produces paired end reads of up to approximately 2 × 300 base pairs, enabling robust taxonomic assignment of hypervariable regions. It is considered flexible for moderate sample sizes typical in poultry microbiome studies, with shorter run times and low error rates (Loman et al., 2012; Di Bella et al., 2013; Saeed and Usman, 2019). Although both Illumina platforms are limited by short read length and relatively long run times, the MiSeq typically has a shorter run time of approximately 4 to 55 hours, compared with the HiSeq, which can require up to about three days to generate full output (Saeed and Usman, 2019). Nevertheless, both platforms are constrained by short read length, and platform choice influences diversity metrics and relative abundance estimates (Luo et al., 2012). Third generation sequencing platforms, including single molecule real time sequencing and nanopore sequencing, require less time for DNA preparation because they do not rely on PCR amplification and are considered cost effective (Kumar and Pitta, 2015). Continued adoption of these rapid and portable technologies is expected to further advance understanding of the poultry gut microbiome.

When using these technologies, poultry-specific considerations must be made at every stage, from experimental design to the choice of sequencing platform. For example, sequencing depth (i.e., the number of reads generated) is a key factor in selecting the appropriate NGS technology, as different platforms offer varying levels of throughput and depth. The required depth of coverage depends on several factors, including the experiment's goal. Studies targeting dominant taxa require lower depth than those aiming for comprehensive community characterization. Sequencing platforms can also impact results, influencing microbial diversity and relative abundance (Luo et al., 2012). Platform selection must therefore consider sequencing yield, cost, workflow complexity, and analytical capacity.

#### Shotgun metagenomics

Although 16S rRNA gene amplicon sequencing has provided extensive information on poultry microbial communities, it suffers the limitations of functional relevance. While amplicon sequencing has enabled poultry researchers to answer the question of “who is there” via taxonomic profiling, shotgun metagenomics enables us to answer the question “what can they do” . Although functional prediction from 16S datasets is also possible, this often relies on statistical inference and reference genome availability. However, poultry associated microorganisms are underrepresented in public databases relative to human microbiota, hence predicted functions from 16S data in poultry require cautious interpretation.

In shotgun metagenomic analysis, total DNA from a sample is sequenced without target specific amplification (Quince et al., 2017). This allows simultaneous identification of bacteria, archaea, viruses, and eukaryotes (Quince et al., 2017; Lind and Pollard, 2021). Shotgun whole genome sequencing supports strain level taxonomic resolution, functional gene annotation, and reconstruction of metagenome assembled genomes (Somerville et al., 2019). The first metagenomic study of the chicken gut microbiome used random pyrosequencing to compare challenged and pathogen free birds, demonstrating the importance of mobile genetic elements in cecal communities and their role in horizontal gene transfer (Qu et al., 2008). The first comprehensive gene catalog of the chicken gut microbiome, covering all intestinal compartments, was created in 2018 using chicken metagenomic data (Huang et al., 2018). Most shotgun sequencing in poultry have been performed using 454 pyrosequencing or Illumina platforms (Danzeisen et al., 2011; Huang et al., 2018; Segura-Wang et al., 2021), although long read systems from Pacific Biosciences and Oxford Nanopore Technologies are known to improve assembly quality and biosynthetic gene cluster reconstruction (Xie et al., 2020).

Shotgun metagenomics overcomes many amplification related biases and enables evaluation of gene content, functional capacity, and intra population heterogeneity (Shah et al., 2011; Scholz et al., 2012). However, it presents technical and analytical challenges. Library preparation requires substantial quantities of high quality DNA, typically around 100 ng for whole genome sequencing libraries. Data analysis is computationally intensive due to the scale and complexity of sequence data, and assembly is complicated by shared genomic regions among related taxa (Sharpton, 2014). Contamination remains a concern, particularly when bioinformatic pipelines do not adequately identify and filter exogenous DNA (Schmieder and Edwards, 2011). Metagenomic sequencing also cannot distinguish DNA from live versus dead organisms and requires careful separation of host and microbial signals (De Cesare et al., 2018). Standardized guidelines for sample size and sequencing depth in poultry shotgun metagenomics are not yet established. Evidence from human studies indicates that adequate sample numbers are required to reliably reconstruct low abundance species and gene sets. For example, sequencing of 396 human stool samples identified low abundance taxa such as *Bifidobacterium animalis* subsp. *lactis* and suggested that approximately 18 samples were required to recover this organism at resolution comparable to the full dataset (Nielsen et al., 2014). However, poultry populations raised under controlled conditions, irrespective of treatments may exhibit lower variability than human cohorts, potentially reducing required sample numbers, but optimal design remains dependent on research objectives and expected community complexity. Development of unified methodological guidelines, especially as it relates to poultry studies would facilitate broader adoption and enable robust inter study comparison.

Beyond functional profiling, shotgun metagenomics supports antimicrobial resistance surveillance (Guardabassi et al., 2019; Humphreys and Coleman, 2019), investigation of foodborne outbreaks (Inns et al., 2017; Jackson et al., 2016), and pathogen tracking through core genome multilocus sequence typing (Köser et al., 2014; Sanabria et al., 2021). The long-term objective of metagenomics is reconstruction of genomes from previously unculturable microorganisms through assembly of overlapping fragments into complete chromosomes (Singh et al., 2008). Although extensive literature describes taxonomic profiling of the poultry gut microbiome, functional and genomic resolution remains comparatively limited. Shotgun metagenomics addresses this gap by integrating composition with metabolic capacity and evolutionary dynamics, thereby advancing mechanistic understanding of host microbe interactions in poultry systems.

#### Application and future directions

NGS technologies, such as Illumina and Oxford Nanopore platforms, have revolutionized microbiome research in poultry, providing detailed insights into the microbial communities that influence poultry health, growth, and disease resistance. However, there is still a gap between applications of such technologies in individual research studies or deployed in the field as a tool for more personalized management strategies in poultry production. To fill that gap, a standardized but also cost-effective workflow must be applied on every single sample (e.g. robot for scaling up microbial DNA extraction and library preparation) to ensure both reproducibility and throughput. Processing the sequencing raw data must be fast, automated and use computational power from the cloud for running robust bioinformatics pipelines embedding a catalog of microbial genomes and reference genes of the chicken gut. The latter has become a critical requirement in the development of microbiome service in the poultry industry. Reaching higher resolution and accuracy for decision-marking can only be achieved with the use of curated catalogs and databases. It requires a structured workflow combining high-quality genome collection, strict metadata control, functional annotation, and continuous validation. High quality genome collection must be from trusted sources with high completeness (> 90%) and low contamination (< 5%). Public genomes can be used but metagenome-assembled genomes (MAGs) from published or own shotgun metagenomic datasets are preferred. This step is critical because many chicken gut microbes are uncultured. Typically, hybrid assemblies (Illumina + Nanopore) produce the highest-quality reference genomes. Dereplication, clustering, taxonomy standardization, functional gene annotation are the other steps needed for building a chicken gut gene catalog. A good, curated database should increase mapping rate by 10-30%, improve strain-level detection, and reduce false positives. This allows better taxonomy assignment, more precise functional quantification, and more accurate resistome analysis.

Altogether, standardized wet lab and in-depth bioinformatics work can help create a microbiome commercial database for rapid microbiome profiling, and evaluate how different factors such as diet, genetics, and environmental factors influence the microbiome and its association with the health, performance, and sustainability of poultry flocks.

Manipulating this microbiome presents unique opportunities to develop strategies that enhance animal resilience against enteric stress, improve intestinal function, optimize nutrient utilization, promote animal welfare, and reduce environmental emissions. By focusing on the functional aspects of the microbiome, researchers can explore the activities of microorganisms and the beneficial metabolic by-products they produce for the host.

Additionally, the removal of Antibiotic Growth Promoters (AGPs) from poultry feed has driven the development of alternative feed additives that support the establishment of functional, stable, and reproducible microbial communities. To achieve this, novel technologies, including omics approaches, are being integrated into both poultry research and commercial applications, with the potential to transform poultry production as these technologies become more affordable. For example, by adopting a multifaceted approach, including *ex vivo, in vitro*, and *in vivo* techniques, precision biotic (**PB**) – based glycans have been developed based on their ability to modulate specific functions of the microbiome, especially those related to short-chain fatty acid (**SCFA**) production and protein metabolism and utilization by the microbiome (Jacquier et al., 2022; Bortoluzzi et al., 2023, 2024, 2025a).

The beneficial microbiome modulation by these glycans have been translated into increased resistance against field outbreaks of coccidiosis (Blokker et al., 2022), infectious bronchitis (Lobo et al., 2023), necrotic enteritis (Bortoluzzi et al., 2025b) in broilers, and smothering in laying hens (Petranyi et al., 2024). Leone and Ferrante (2023) reported that, as opposed to conventional prebiotics extracted from yeasts, for example, glycan-based PB are less impacted by external factors and may be used to also improve welfare, litter, and air quality of poultry houses.

We are advancing toward a new frontier in precision microbiome modulation, enabled by our expanding mechanistic understanding and technological capabilities. These advancements encompass, but are not limited to, the development of high-throughput glycan screening platforms, the standardization of robust and reproducible sequencing workflows, the establishment of avian-specific microbiome reference databases, and the implementation of advanced computational tools for multi-dimensional data integration and exploration.

### Deep learning models for understanding microbial communities

Mahalingam Ramkumar (ramkumar@cse.msstate.edu)

Computer Science & Engineering, Mississippi State University, Starkville, MS 39762, USA

Microbiomes, the complex communities of microorganisms residing in the gut, intestines, and other environments, serve as vital indicators of health in humans and animals. Traditional microbiome studies often focus on characterizing alpha diversity (the richness and evenness of microbial taxa within a single sample) and beta diversity (the similarity or dissimilarity in microbial composition among samples). However, these approaches alone may not fully capture the dynamic interactions between microbes, pathogens, and their hosts.

Our study adopts a combinatorial approach, integrating advanced machine learning techniques with 16S rRNA gene sequencing microbiome data to predict the presence of zoonotic food-borne pathogens, Salmonella, Campylobacter, and Listeria, in pastured poultry systems. Additionally, we investigate the role of microbiota in modulating pathogen prevalence, and the emergence of antibiotic resistance, a growing global health concern. Our dataset includes soil and fecal samples collected from 42 distinct poultry farms across the southeastern United States over a six-year period, obtained at multiple stages of poultry development. To contextualize the microbial data, detailed farm management practices were also recorded.

Using this rich dataset, we developed predictive models to identify "ideal" microbiome communities tailored to specific objectives, such as minimizing antimicrobial resistance (AMR), reducing pathogen prevalence, and the effect of different farm practices on abundance of important genera like known probiotics. The results demonstrated that Microbiome composition could predict prevalence of pathogens with greater than 80% accuracy. Significant negative correlation was observed between known probiotics like Bacillus and Clostridium and Campylobacter at mid growth stage. No significant association was found between known probiotics and Salmonella or Listeria. Our models also indicated that certain far management practices (like rotating back to previously used farm lots, moving pasture every other day instead of daily, keeping brood and pasture feeds free from soy products, but with GMO content, and not having other animals on the farm) can boost probiotics abundance and help reduce Campylobacter prevalence.

Microbiome datasets typically suffer from the curse of dimensionality. In other words, the number of features - for example, OTUs - are substantially larger than the number of samples.

One strategy to address this imbalance is by using Twin-regression models, which can turn a dataset with feature dimension d and sample size N to a dataset with feature dimension 2d and Sample dimension N2 (for example, from *d* = 100,*N* = 100 to *d* = 200 and *N* = 10,000). Such a model, when used with the same dataset, indicated that higher abundance of Lactobacillus reduces multi-drug resistance (MDR) in Campylobacter, Salmonella and Listeria. Bacillus class Streptococcaceae and Planococcaceae was correlated with increased MDR in Salmonella and Listeria, but had no effect on Campylobacter. Additionally, Acinetobacter was found to increase MDR in Listeria. The Twin regression model was also used as a foundation for a Bayesian Guided approach to generate ideal combinations of Microbiomes to minimize pathogenicity. From our findings, high abundance of bacteroides, firmicutes, and Ruminococcaceae were found to be associated with pathogen suppression, and even low abundance of Chlamydiaceae plays a critical role in reducing pathogen prevalence.

Unlike traditional ML approaches, some deep learning approaches do not consider dimensionality as a curse. In fact, dimensionality may even be a blessing that provides different views of every sample, thereby providing useful insights. A Graph Neural Network (GNN) model was used to identify microbiome signatures associated with pathogen prevalence. Our models indicated that Tenericutes as a significant influence on prediction models for all 3 pathogens, and that Geobacter and Actinobacteria were the top influencers for models for predicting prevalence of Salmonella and Campylobacter.

In traditional ML models, given a dataset with features x1, x2, …, xn, and label y, the model does not care if x1 is a temperature, or pressure, or fold change in gene expression. Models are simply immune to the semantics of the features. Deep Learning models like Transformers and Graph Neural networks can take advantage of known semantics of such features, possibly learnt from data that has nothing to do with the dataset under analysis. While it is true that deep learning models need a lot more data to be useful, all data need not be from the same experiment / trial.

Deep learning models can uncover complex patterns in microbial interactions that are not readily apparent through traditional statistical methods, or even conventional machine learning approaches. Unlike conventional machine learning frameworks, which often assume that input features (e.g., microbial taxa or environmental variables) are uncorrelated, our approach incorporates more sophisticated deep learning architectures, such as graph neural networks and transformers, that . These models can integrate prior knowledge of feature associations, including those derived from external or seemingly unrelated datasets, to enhance predictive accuracy and biological insight. More importantly, while for conventional machine learning approaches high feature dimensionality is seen as a curse, deep learning approaches can even regard high feature dimensionality as a blessing.

Our current research is focused on constructing large-scale microbiome models, analogous to large language models in natural language processing. These models are designed to encapsulate the vast complexity of microbiome data across diverse ecosystems and hosts. By fine-tuning these models with a small number of targeted samples, we aim to address specific research questions, such as identifying microbial signatures of health or disease. Furthermore, these advanced modeling techniques facilitate the development of more explainable models, which can elucidate the biological mechanisms underlying microbiome-pathogen interactions. This explainability is critical for translating microbiome research into practical applications, such as designing probiotic interventions or informing farm management practices to enhance animal health and food safety.

Our work has the potential to transform microbiome research by providing a scalable, adaptable framework for studying microbial communities. By harnessing the power of deep learning and large-scale data integration, we aim to unlock new insights into the role of microbiomes in health, disease, and environmental sustainability, with implications for agriculture, veterinary medicine, and public health.

#### Case Studies: Biological interpretation and inference from microbiome datasets using machine learning

Our series of studies (Ayoola, Pillai et al., 2023; Pillai, Nanduri et al., 2023, Nanduri et al., 2024; Ram Das, Pillai et al., 2024) illustrate how ML can move microbiome research beyond descriptive profiling to uncover biologically meaningful patterns that inform pathogen control and farm management strategies. Applying ML to pastured poultry-related microbiome enabled the prediction of prevalence of foodborne pathogens and probiotics (Ayoola, Pillai et al., 2023). Our results demonstrate that pathogen prevalence in pastured poultry is governed by community-level microbial interactions rather than individual taxa alone. ML models revealed that known probiotics, such as Bacillus and Clostridium, negatively correlate with Campylobacter, suggesting roles for competitive exclusion or metabolic antagonism, particularly during mid-growth stages when pathogen prevalence is highest. By contrast, there was no significant association between known probiotics and Salmonella or Listeria, indicating pathogen-specific ecological relationships within the microbial community rather than generalized probiotic effects. Temporal dynamics were also evident, as the strength of the predicted relationships between probiotics and Campylobacter varied across growth stages (START, MID, END), highlighting that microbiome-pathogen interactions are not static but evolve during host development. In addition, the study identified associations between specific farm management practices-such as frequency of pasture house movements, age at pasture introduction, diet composition (e.g., soy-free or GMO-free feed), and the presence of other animals on the farm and the abundance of probiotic taxa, illustrating how environmental and operational factors shape microbial ecology. This work illustrates that ML can uncover mechanistic insights from complex microbiome datasets, providing a foundation for targeted interventions such as probiotic supplementation or management strategies to reduce foodborne pathogens and promote poultry gut health.

We extended our analyses and the ecological interpretation of microbiome data to AMR phenotypes among major foodborne pathogens. Using regression and association models, we found that increased relative abundance of Lactobacillus was significantly correlated with reduced multidrug resistance in Campylobacter, Salmonella, and Listeria isolates (Pillai, Nanduri et al., 2023), consistent with the established role of Lactobacilli in competitive exclusion and inhibition of pathogenic traits. By contrast, taxa such as members of Bacillus-related families and Acinetobacter were associated with elevated MDR phenotypes, suggesting that some community constituents may facilitate resistance gene maintenance or horizontal gene transfer within the microbiome. These patterns point to a functional link between microbial community composition and emergence of resistance indicating that AMR phenotypes are not independent of community ecology and reflect the integrated behavior of microbial networks. Furthermore, these associations underscore that manipulating microbiome structure through management practices could modulate resistance risk, offering a biologically informed route to mitigate AMR in agricultural systems.

With Bayesian-Guided Generation of Synthetic Microbiomes (Pillai, Nanduri et al., 2024), we moved from descriptive and predictive modeling to an ecologically informed generative framework. By coupling Bayesian inference with microbiome composition profiles, we identified synthetic microbiome configurations predicted to minimize pathogen prevalence and suppress virulence traits. This approach revealed that not only dominant taxa (e.g., broad Firmicutes and Bacteroidetes) but also lower-abundance taxa contributed meaningfully to predicted community behavior, highlighting that taxa with modest relative abundance may still exert disproportionate ecological influence. Although these models are based on probabilistic modeling rather than simple correlation, the biological inference remains clear: pathogen suppression and resistance mitigation arise from coordinated interactions within the microbial community. In practice, such generative microbiome predictions provide insight into how ecological balance, involving both abundant and rare taxa, can promote ecosystem stability and reduce pathogen risk in complex host environments.

Using Transformer-Based Models and Attention Explainability, deep learning approaches were applied to identify complex, higher-order relationships among microbial taxa that influence pathogen prevalence (Ram Das, Pillai et al., 2024). Transformer models equipped with attention mechanisms revealed interpretable patterns of microbial co-occurrence and context-dependent interactions, highlighting that pathogen presence is shaped by structured ecological networks rather than individual taxa in isolation. These models enabled the detection of taxa whose relative abundance and associations with other community members were particularly informative for predicting pathogen outcomes. Moreover, attention-based interpretability provides a framework for linking predictive performance to ecologically meaningful microbial interactions, supporting informed interventions in poultry farm management.

**Fig. 2:**
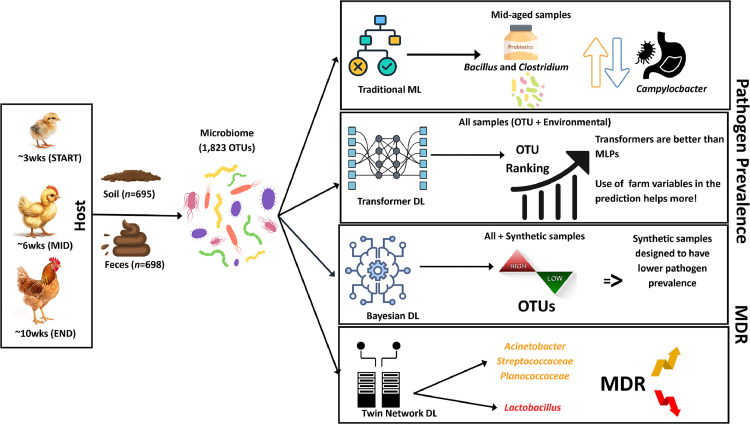
Integrated machine learning frameworks reveal ecological drivers of pathogen prevalence and multidrug resistance in longitudinal poultry microbiomes. Schematic overview of four complementary studies analyzing 1,823 microbial OTUs derived from soil (*n* = 695) and fecal (*n* = 698) samples collected from pastured poultry across three growth stages (∼3 weeks, START; ∼6 weeks, MID; ∼10 weeks, END). Microbiome profiles were analyzed using traditional machine learning (ML), transformer-based deep learning (DL), Bayesian DL, and twin-network DL architectures to uncover biologically meaningful patterns associated with pathogen prevalence and multidrug resistance (MDR).

A schematic overview of four complementary studies analyzing 1,823 microbial OTUs derived from soil (*n* = 695) and fecal (*n* = 698) samples collected from pastured poultry across three growth stages (∼3 weeks, START; ∼6 weeks, MID; ∼10 weeks, END) is presented Fig. 2. Microbiome profiles were analyzed using traditional machine learning (ML), transformer-based deep learning (DL), Bayesian DL, and twin-network DL architectures to uncover biologically meaningful patterns associated with pathogen prevalence and multidrug resistance (MDR). In mid-aged samples, traditional ML identified negative associations between probiotic genera (*Bacillus* and *Clostridium*) and *Campylobacter*, particularly during the mid-growth stage when pathogen prevalence was highest, suggesting competitive exclusion or inhibitory microbial interactions. Transformer-based DL applied to all samples (including OTU and environmental/farm variables) improved pathogen prediction performance compared to multilayer perceptrons (MLPs) and enabled OTU ranking, demonstrating that incorporating farm management variables enhances predictive accuracy.

Bayesian-guided modeling extended these analyses by generating synthetic microbiome configurations predicted to exhibit lower pathogen prevalence, illustrating that pathogen suppression reflects coordinated community-level dynamics rather than single-taxon effects.

Twin-network DL models linked microbiome composition to MDR phenotypes, identifying taxa such as *Acinetobacter, Streptococcaceae*, and *Planococcaceae* as positively associated with elevated MDR, whereas Lactobacillus abundance correlated with reduced resistance. Collectively, the figure highlights that pathogen prevalence and antimicrobial resistance are shaped by structured microbial networks, temporal host development, and environmental factors. By integrating predictive, generative, and interpretable ML approaches, these studies demonstrate how complex microbiome datasets can be translated into ecologically coherent insights to inform farm management and microbiome-based intervention strategies.

Together, these results illustrate how integrating predictive, generative, and interpretable ML frameworks can yield ecologically coherent insights, translating complex microbiome data into actionable understanding of pathogen ecology, AMR modulation, and potential microbiome-informed interventions.

## Conclusions

Microbiome research inquiry in poultry science is expanding rapidly, bringing with it a wealth of new opportunities and analytical tools. To fully capitalize on this growth and the possibilities within the field, researchers must prioritize the use of appropriate tools (such as multi-omic approaches), select the right sampling sites (e.g., invasive vs. cloacal/fecal), standardize protocols, adopt field-uniform bioinformatics pipelines, and implement standardized, cost-effective NGS workflows. These measures will facilitate reproducibility and consistency across studies. In applying NGS technology to poultry microbiota profiling, the field must move beyond simple microbiota censuses (“Who is there?”) towards advanced functional microbiome analyses (“What are they doing?”). This requires the integration of multi-omic technologies, including amplicon sequencing, metagenomics, and metatranscriptomics, as no single approach fits all research questions. Adopting artificial intelligence, machine learning, and deep learning could also further accelerate progress within the field. Deep learning, in particular, offers distinct advantages for microbiome analysis by uncovering complex patterns of microbial interactions that are not readily detected by traditional statistical methods, integrating prior knowledge of feature associations, and incorporating biological insights. These capabilities highlight the potential of deep learning and large-scale data integration to transform microbiome research.

Another important issue that requires attention is the revalidation of microbiota results obtained from amplicon-based sequencing or metagenomics using qPCR methods. Bioinformatic and statistical approaches could potentially overestimate relative abundances. Similar to other fields, the microbiome community should prioritize revalidating bacterial thresholds, particularly for taxa with available primers or culturable strains. Relative abundances should not be labeled as 'high' or 'low' based solely on statistical analysis; they should be revalidated and interpreted through biological and metabolic insights, when possible. Continued development of robust, scalable workflows and avian-specific reference databases will be critical for translating microbiome discoveries into practical applications that enhance poultry health, productivity, and sustainability.

## CRediT authorship contribution statement

**Samson Oladokun:** Writing – review & editing, Writing – original draft, Supervision, Conceptualization. **Bertrand Grenier:** Writing – review & editing, Writing – original draft, Investigation. **Brian Oakley:** Writing – review & editing, Writing – original draft, Investigation. **Cristiano Bortoluzzi:** Writing – review & editing, Writing – original draft, Investigation. **Mahalingam Ramkumar:** Writing – review & editing, Writing – original draft, Investigation.

## Disclosures

The authors have no conflict of interests. Fig. 1 was generated with the assistance of AI (NotebookLM, Google) and information adapted from cited sources.
